# Supplementary material for: Infection of Rhesus Macaques with O’nyong-nyong virus UVRI-0804 recapitulates key aspects of human clinical disease
Source: PLoS Negl Trop Dis. 2026 Jul 10;20(7):e0013780. doi: 10.1371/journal.pntd.0013780 (PMC13436782; doi:10.1371/journal.pntd.0013780)
Supplement: Supplementary file 2 [file pntd.0013780.s001.docx]

|  |  | **ONV 1** | **ONV 2** | **ONV 3** | **ONV 4** | **ONV 5** | **ONV 6** |
| --- | --- | --- | --- | --- | --- | --- | --- |
|  | % vRNA Tissue | 38/77 (49.35%) | 19/80 (23.75%) | 11/80 (13.75%) | 8/81 (10%) | 30/83 (36.14%) | 35/82 (42.68%) |
| **Lymphoid Tissue** | Axillary LN | 5.87 | — | 3.67 | 2.68 | 5.81 | 4.89 |
|  | Cervical LN | 3.59 | 2.80 | 2.68 | — | — | 4.03 |
|  | Inguinal LN | — | 3.53 | 3.47 | 3.14 | 2.64 | 4.75 |
|  | Mesenteric LN | — | — | — | — | 2.29 | 3.17 |
|  | Popliteal LN | — | 3.70 | 3.76 | — | — | 4.76 |
|  | Retroperitoneal LN | — | — | — | — | — | — |
|  | Salivary LN | 3.06 | — | 4.58 | — | — | 4.50 |
|  | Spleen | 5.26 | 4.15 | — | 3.58 | 4.45 | — |
|  | Thymus | — | — | — | — | 2.43 | — |
|  | Tonsil | — | — | — | 2.33 | 3.86 | — |
| **Musculoskeletal** | | | | | | | |
| **Joints** | L. Ankle | 2.71 | 2.17 | — | — | — | — |
|  | R. Ankle | — | 2.59 | — | — | 2.31 | — |
|  | L. Elbow | — | 2.38 | — | — | 2.46 | 2.33 |
|  | R. Elbow | 2.45 | — | — | — | — | 2.52 |
|  | L. Finger | 4.76 | 4.03 | 2.76 | 3.52 | — | — |
|  | R. Finger | 3.42 | 3.36 | 3.45 | 2.87 | 3.76 | 2.52 |
|  | L. Knee | 3.68 | — | — | — | — | — |
|  | R. Knee | 2.88 | — | — | — | 3.08 | 3.16 |
|  | L. Toe | 3.15 | — | — | — | — | 3.30 |
|  | R. Toe | 3.86 | — | — | — | — | 3.15 |
|  | L. Wrist | 4.14 | 2.78 | — | — | 4.38 | 2.84 |
|  | R. Wrist | 4.25 | 3.38 | — | — | 3.36 | 2.75 |
|  | Synovial Fluid Wrist |  | 2.67 |  | 4.22 | — | — |
|  | Synovial Fluid Elbow |  |  |  |  | — |  |
|  | Synovial Fluid Ankle |  |  | — | — | — | — |
| **Muscles** | L. Biceps Brachii | — | — | — | — | — | — |
|  | R. Biceps Brachii | — | — | — | — | — | — |
|  | L. Brachioradialis | — | — | — | — | — | — |
|  | R. Brachioradialis | — | — | — | — | — | — |
|  | L. Hamstring | 2.26 | — | — | — | — | 2.70 |
|  | R. Hamstring | 2.56 | — | — | — | — | — |
|  | L. Quadriceps | — | — | — | — | 2.50 | — |
|  | R. Quadriceps | — | — | — | — | 2.49 | — |
|  | L. Soleus | 2.55 | — | — | — | 3.19 | 3.00 |
|  | R. Soleus | — | — | — | — | 2.36 | — |
|  | L. Triceps Brachii | 3.22 | — | — | — | 3.55 | 2.43 |
|  | R. Triceps Brachii | 2.56 | — | — | — | — | — |
| **Skin** | Skin Upper Torso | 2.20 | — | — | — | — | — |
|  | Rash Abdomen |  |  |  | — |  |  |
|  | L. Axillary Rash |  |  | 2.13 |  | 3.23 | — |
|  | L. Wrist Skin Rash |  | 2.52 | 4.29 |  | 3.32 | — |
|  | R. Wrist Skin Rash |  | 3.80 |  |  | 3.21 | 2.78 |
|  | Lip Skin Lesion |  |  |  | 2.45 |  |  |
| **Major Organ Systems** | | | | | | | |
| **Cardiopulmonary and Renal Tissues** | Heart | 3.04 | — | — | — | — | — |
|  | Lung | — | — | — | — | 3.14 | 2.17 |
|  | Kidney | 3.31 | — | — | — | 2.94 | — |
|  | Ureter | 2.98 | — | — | — | — | 3.62 |
|  | Urethra | 3.12 | — | — | — | — | 2.33 |
|  | Urinary Bladder | — | 2.45 | — | — | — | — |
| **Digestive Tissues** | Parotid Gland | — | — | — | — | — | 2.23 |
|  | Submandibular Gland | — | — | — | — | 2.32 | — |
|  | Esophagus | — | — | — | — | 3.27 | — |
|  | Stomach | 2.25 | — | — | — | — | — |
|  | Duodenum | — | — | — | — | — | — |
|  | Jejunum | 3.30 | — | — | — | — | — |
|  | Ileum | — | — | — | — | — | — |
|  | Colon | 2.56 | — | — | — | — | — |
|  | Liver | — | — | — | — | — | 3.55 |
|  | Gallbladder | 2.41 | — | — | — | — | — |
|  | Pancreas | — | — | — | — | — | 2.49 |
| **Reproductive Tissue** | Ovaries | 2.76 | — | — |  |  |  |
|  | Uterus | — | — | — |  |  |  |
|  | Cervix | 2.52 | — | — |  |  |  |
|  | Fallopian Tube | 2.25 | — | — |  |  |  |
|  | Vagina | — | — | — |  |  |  |
|  | Seminal Vesicles |  |  |  | — | 2.22 | — |
|  | Testes |  |  |  | — | — | — |
|  | Epididymis |  |  |  | — | — | — |
|  | Prostate |  |  |  | — | — | — |
|  | Vas Deferens |  |  |  | — | — | — |
| **Endocrine Tissues** | Adrenal Gland | 2.39 | — | — | — | — | — |
|  | Mammary Gland | 2.50 | — | — | — | — | 2.73 |
|  | Thyroid Gland | 2.87 | 2.24 | 2.64 | — | — | 2.58 |
| **Nervous Tissue** | | | | | | | |
| **Central Nervous System** | Cerebral Spinal Fluid | — | — | — | — | — | — |
|  | Cervical Spinal Cord | 3.50 | — | — | — | — | 3.15 |
|  | Thoracic Spinal Cord | 2.47 | — | — | — | 2.25 | — |
|  | Lumbar Spinal Cord | — | — | — | — | — | 3.76 |
|  | Brain stem | 2.38 | — | — | — | — | — |
|  | Cerebellum | — | — | — | — | 2.65 | — |
|  | Frontal lobe | — | — | — | — | — | 2.21 |
|  | Occipital Lobe | — | — | — | — | — | 2.52 |
|  | Parietal Lobe | — | 3.33 | — | — | — | 2.70 |
|  | Temporal lobe | — | — | — | — | — | 2.14 |
|  | Pituitary Gland | — | — | — | — | 2.68 | 2.35 |
|  | Basal Ganglia | — | — | — | — | — | — |
|  | Eyes | — | — | — | — | — | — |
| **Peripheral Nervous System** | Brachial Plexus | — | 3.33 | — | — | — | 3.15 |
|  | Dorsal Root Ganglion | — | — | — | — | 2.37 | — |
|  | Femoral nerve | — | 3.10 | — | — | — | — |
|  | Sciatic Nerve | 2.51 | — | 3.63 | — | 2.78 | 4.17 |
|  | Trigeminal Nerve | — | — | — | — | — | — |

**S1 Table ONNV Tissue Distribution**
