# Supplementary material for: Infection of Rhesus Macaques with O’nyong-nyong virus UVRI-0804 recapitulates key aspects of human clinical disease
Source: PLoS Negl Trop Dis. 2026 Jul 10;20(7):e0013780. doi: 10.1371/journal.pntd.0013780 (PMC13436782; doi:10.1371/journal.pntd.0013780)
Supplement: Supplementary file 3 [file pntd.0013780.s002.docx]

| **Grade** | **Scoring Parameter** |
| --- | --- |
| 0 | No inflammation |
| 1 to 2 | Sparse (focal to multifocal perivascular lymphocytic aggregates, one layer, affecting ≤3 vessels) |
| 3 to 4 | Minimal (multifocal perivascular lymphocytic aggregates, one to three layers, affecting >3 vessels) |
| 5 to 6 | Mild (multifocal perivascular lymphocytic infiltrates forming ≥4 layers, affecting >6 vessels) |
| 7 to 8 | Moderate (multifocal perivascular lymphocytic infiltrates and inflammation in multiple tissue types) |
| 9 to 10 | Severe (multifocal perivascular lymphocytic infiltrates, perivasculitis, inflammation +/- tissue destruction in multiple tissue types) |

**S2 Table Histologic grading rubric of inflammation in the joints, muscle, and skin of ONNV-infected rhesus macaques.**
