# Supplementary material for: Infection of Rhesus Macaques with O’nyong-nyong virus UVRI-0804 recapitulates key aspects of human clinical disease
Source: PLoS Negl Trop Dis. 2026 Jul 10;20(7):e0013780. doi: 10.1371/journal.pntd.0013780 (PMC13436782; doi:10.1371/journal.pntd.0013780)
Supplement: Supplementary file 4 [file pntd.0013780.s003.docx]

|  | **Joints** | | | | | | | | | | | | | | | | | | | | **Skin** | | |
| --- | --- | --- | --- | --- | --- | --- | --- | --- | --- | --- | --- | --- | --- | --- | --- | --- | --- | --- | --- | --- | --- | --- | --- |
|  | **Elbow** | | | **Wrist joint** | | | **Metacarpal- Interphalangeal** | | | **Knee** | | | **Ankle** | | | | **Metatarsal- Interphalangeal** | | | | **Rash** | | |
| ID | T | M | C | T | M | C | T | M | C | T | M | C | T | M | C | T | | M | C | T | | M | C |
| ONV1 | 3 | 0 | 0 | 4 | 0 | 1 | 4 | 1 | 0 | 3 | 0 | 0 | 2 | 3 | 1 | 3 | | 1 | 1 |  | |  |  |
| ONV2 | 3 | 0 | 1 | 4 | 4 | 6 | 6 | 6 | 8 | 2 | 0 | 0 | 2 | 0 | 0 | 3 | | 2 | 1 | 5 | | 4 | 7 |
| ONV3 | 4 | 0 | 0 | 3 | 3 | 2 | 6 | 4 | 3 | 2 | 0 | 0 | 2 | 1 | 0 | 4 | | 1 | 0 | 6 | | 5 | 7 |
| ONV4 | 4 | 0 | 1 | 6 | 4 | 5 | 6 | 5 | 8 | 3 | 0 | 2 | 1 | 1 | 0 | 3 | | 1 | 0 | 6 | | 4 | 3 |
| ONV5 | 3 | 1 | 3 | 2 | 0 | 1 | 5 | 4 | 5 | 3 | 1 | 1 | 3 | 3 | 2 | 2 | | 4 | 4 | 7 | | 2 | 7 |
| ONV6 | 3 | 0 | 0 | 4 | 5 | 5 | 2 | 5 | 7 | 2 | 0 | 0 | 4 | 1 | 0 | 4 | | 1 | 2 | 4 | | 2 | 5 |

**S3 Table Histologic scores from evaluated joints and rashes.**
