# Supplementary material for: Infection of Rhesus Macaques with O’nyong-nyong virus UVRI-0804 recapitulates key aspects of human clinical disease
Source: PLoS Negl Trop Dis. 2026 Jul 10;20(7):e0013780. doi: 10.1371/journal.pntd.0013780 (PMC13436782; doi:10.1371/journal.pntd.0013780)
Supplement: Supplementary file 5 [file pntd.0013780.s004.docx]

**S4 Table Virus information**

| **Virus** | **Strain** | **Isolation** | **Region** | **Resource** |
| --- | --- | --- | --- | --- |
| Mayaro virus | BeAr505411 | 1991 | Brazil | BEI NR-49910 |
| Una virus | MAC150 | 1997 | Venezuela | BEI NR-49912 |
| O’nyong nyong virus | UgMP30 | 1959 | Uganda | BEI NR-51661 |
| O’nyong nyong virus | 0804 | 2017 | Uganda | PMID: 36326501  PMID: 40096126 |
| Ross River virus | T-48 | 1959 | Australia | BEI NR-51457 |
| Semliki forest virus | Dakar B 16878 | 1980 | Central African Republic | WRCEVA TVP 21326 |
| Venezuelan equine encephalitis virus | TC-83 | 1961 | Trinidad | BEI NR-63 |
| Madariaga virus | PAN 243178 | 2010 | Panama | WRCEVA TVP 5053 |
